# The Effect of Macronutrient Availability on Pomegranate Reproductive Development

**DOI:** 10.3390/plants9080963

**Published:** 2020-07-30

**Authors:** Silit Lazare, Yang Lyu, Uri Yermiyahu, Yehuda Heler, Gershon Kalyan, Arnon Dag

**Affiliations:** 1 Gilat Research Center, Agricultural Research Organization, The Volcani Center, M.P. Negev 85280, Gilat, Israel; lyu.yang.china@gmail.com (Y.L.); uri4@volcani.agri.gov.il (U.Y.); yehuda.heler@mail.huji.ac.il (Y.H.); arnondag@volcani.agri.gov.il (A.D.); 2 College of Resources and Environmental Sciences, China Agricultural University, Beijing 100193, China; 3 Institute of Plant Sciences and Genetics in Agriculture, The Robert H. Smith Faculty of Agriculture, Food and Environment, The Hebrew University of Jerusalem, Rehovot 7628604, Israel; 4 Fertilizers and Chemicals Ltd., Haifa 12949, Israel; Gershon.Kalyan@icl-group.com

**Keywords:** andromonoecy, flowering, male flower, bisexual flower, NPK, *Punica granatum*

## Abstract

Pomegranate cultivation has expanded significantly in the last two decades. However, there is limited information on its fertilization requirements and the effect of macronutrient availability on its reproductive development. Two commercial pomegranate cultivars—“Wonderful” and “Emek”—were grown in 500-L containers for 3 years, using a fertigation system. Development and reproduction indices were measured to explore the trees’ responses to elevated levels of nitrogen (N), phosphorus (P) and potassium (K) in the irrigation solution. Andromonoecy rate was affected by nutrient levels only in the first year of the experiment, with higher levels of N and P leading to a greater proportion of hermaphrodites out of total flowers. P level had a positive effect on the total number of hermaphrodites per tree in both varieties. Differences recorded between hermaphroditic and staminate flowers included nutrient concentrations and dry weight. Fruit set and aril number were positively affected by N concentration in the irrigation solution. We conclude that only a severe deficiency of N and P affects the andromonoecy trait, and that at the levels examined in this study, K hardly influences pomegranate reproduction.

## 1. Introduction

About 2% of known plant species are andromonoecious—a floral dimorphic sexual system in which an individual plant bears both hermaphroditic (bisexual) and staminate (male) flowers [1]. Several evolutionary explanations for andromonoecy have been suggested. The resource reallocation hypothesis presumes that the development of male flowers has a lower energy cost than the development of bisexuals, and the energy saved by the mass production of staminate flowers might be reallocated to hermaphroditic flower development and fruit set [2,3]. Another hypothesis is that male flowers are more attractive to pollinating insects, and andromonoecy increases the chances of pollinators reaching these flowers and carrying the pollen [4]. However, to set fruit, a specific plant must attract insects to the female organs as well (otherwise there is no fertilization). Because pollinators are more attracted to large flowers [5], namely hermaphrodites rather than staminate flowers, this hypothesis is evolutionarily problematic. Nevertheless, when energy is scarce, spreading genes through pollen is a reasonable solution. Hand pollination of cashew stigmas with pollen from hermaphroditic or staminate flowers revealed a significant advantage for the male flowers in terms of grain germination and penetration into the ovule [6]. A similar study in *Solanum carolinense* revealed that pollen grains from male flowers are more vigorous than those from bisexual ones [7]. These results probably reflect the nutritional advantage of staminate flower organs over those of hermaphrodites, due to a lack of resource competition [8]. The ratio of bisexual to male flowers was studied in mango and olive trees, and was found to vary depending on the cultivar, growth conditions (climate, irrigation), and the previous season’s production [9,10].

Pomegranate is an andromonoecious plant, defined by large hermaphroditic flowers along with smaller staminate ones, containing the remains of degenerated female organs [11]. The staminate flowers typically fail to set fruit [12]. The pomegranate tree is adapted to semiarid regions, and cultivated mostly in the USA, the Mediterranean basin, and Asia [13]. Pomegranate productivity is of high economic value, its fruits are considered nutraceuticals [14], and the crop is gaining in popularity among growers and consumers [15]. The level of pomegranate andromonoecy (ratio of bisexual to male flowers) varies among genotypes and growth conditions, and staminate flowers might reach 70% of the total flowers on a tree [16]. Studies on the impact of mineral nutrition on pomegranate growth and productivity were recently published [17,18], but they focused mainly on nitrogen (N), and did not refer to flower type.

As staminate flowers are considered to bear better pollen, and because nutrients affect pollen quality in general [19], our hypothesis was that the nutritional status of the tree might influence the hermaphrodite-to-staminate flower ratio and the related reproductive parameters to compensate between the two. To test this, we supplied several concentrations of N, phosphorus (P), and potassium (K) to pomegranate trees and monitored their reproductive development over 3 years. We hypothesized that, under nutrient deficiency, the plant will produce more staminate flowers so as to balance the number of developing fruits according to its resources. The objective of the study was to elucidate the effect of mineral levels on andromonoecy indices in pomegranate. To the best of our knowledge, no previous study has been published in this context.

## 2. Results

**Figure 1:**
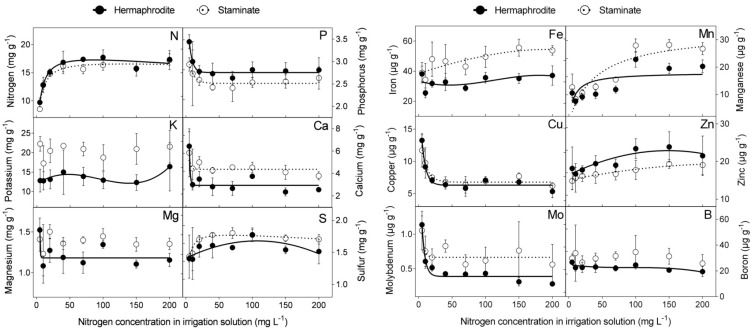
Macro- and microelement concentrations in hermaphroditic and staminate flowers of “Wonderful” pomegranate trees, as a function of N concentration in the irrigation solution, in 2018. Bars represent standard deviation (SD). Lines represent best-fit regression. Regression line equations and significance between treatments are presented in Table S1. No regression line means that no significant difference (*p* < 0.05) was found between the treatments.

The effect of N level in the irrigation solution (5, 10, 20, 40, 70, 100, 150, and 200 mg L^−1^) was examined in macro- and microelement concentrations in the flowers of cv. “Wonderful” trees. N concentrations in hermaphroditic and staminate flowers were similar. Increasing N levels in the irrigation solution, up to 40 mg L^−1^, increased N concentration in the flowers, which leveled off at higher N levels. The concentrations of sulfur (S), iron (Fe), manganese (Mn), and zinc (Zn) increased with N level in the irrigation solution, whereas a negative impact was recorded for P, calcium (Ca), copper (Cu), and molybdenum (Mo) concentrations in both flower types. N levels had no significant impact on K, magnesium (Mg), or boron (B) concentrations in staminate flowers. At most N levels tested, staminate flowers of “Wonderful” trees had significantly higher concentrations of K, Ca, Fe, Mg, Mn, and Mo compared to hermaphroditic ones (Figure 1). None of the nutrient contents was significantly higher in hermaphrodites.

**Figure 2:**
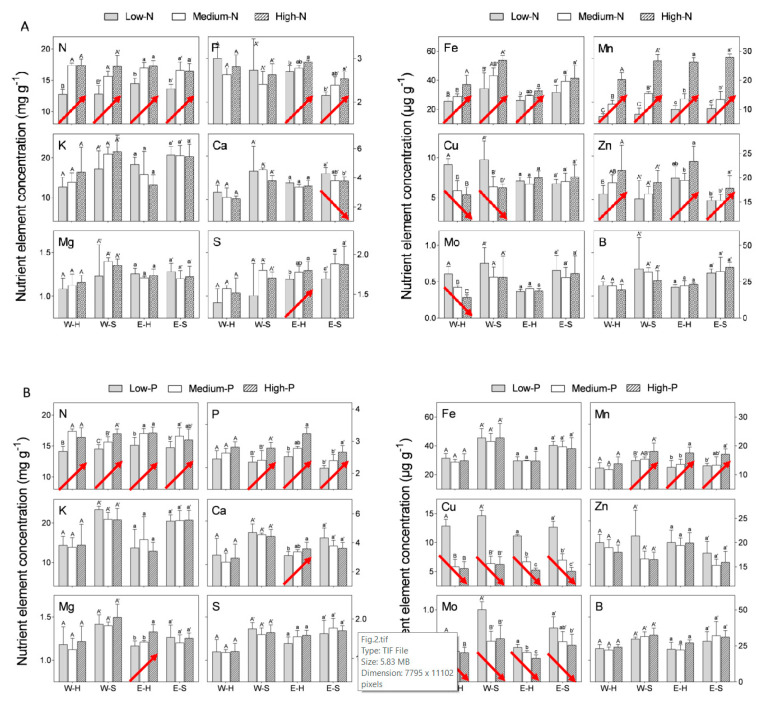

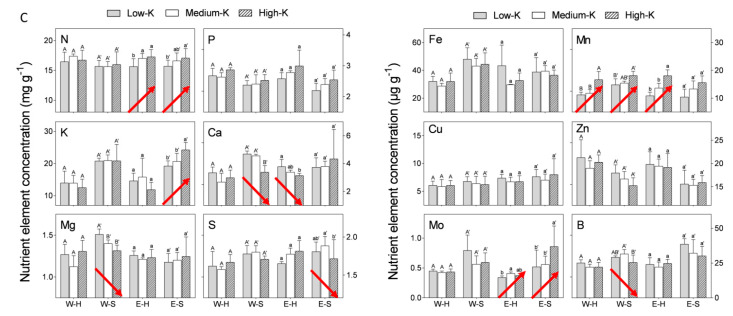
Macro- and microelement concentrations in hermaphroditic and staminate flowers of “Emek” and “Wonderful” pomegranate trees, as a function of (**A**) N, (**B**) P, and (**C**) K levels in the irrigation solution, in 2018. Different letters represent significant differences (*p* ≤ 0.05) between treatments. Significant trends are marked with an arrow. W-H and W-S, “Wonderful” hermaphroditic and staminate flowers, respectively; E-H and E-S, “Emek” hermaphroditic and staminate flowers, respectively.

We compared “Emek” and “Wonderful” trees subjected to three different N levels in the irrigation solution (see Section 4.1 for overall composition of the irrigation solution): low (10 mg N L^−1^ for “Wonderful” and 20 mg N L^−1^ for “Emek”), medium (70 mg N L^−1^ for both cultivars) and high (200 mg N L^−1^ for “Wonderful” and 100 mg N L^−1^ for “Emek”). All three N levels positively affected the N concentration in both hermaphroditic and staminate flowers of both cultivars (Figure 2A). Mn and Fe concentrations responded similarly. N levels in the irrigation solution also positively affected P and Zn concentrations in both flower types of “Emek”, and Zn concentration in hermaphrodites of “Wonderful”. N levels negatively affected Cu concentration in both flower types of “Wonderful”, and Mo concentration in hermaphrodites of this cultivar.

We then compared “Emek” and “Wonderful” trees subjected to three different P levels in the irrigation solution: low (1 mg P L^−1^), medium (10 mg P L^−1^), and high (20 mg P L^−1^) (levels were the same for both cultivars). An increasing P level in the irrigation solution positively affected P concentration in both hermaphroditic and staminate flowers, for both cultivars (Figure 2B). N and Mn responded similarly. P level also positively affected Ca and Mg concentrations in hermaphrodites of “Emek”, but negatively affected Cu and Mo concentrations in both flower types of both cultivars.

An increasing K level (low: 20 mg K L^−1^, medium: 100 mg K L^−1^, high: 200 mg K L^−1^) in the irrigation solution positively affected only K concentration in “Emek” staminate flowers (Figure 2C). N and Mo concentrations were positively affected in both hermaphroditic and staminate flowers of “Emek”, and Mn concentration was positively affected in both flower types of both cultivars (Figure 2C). K level negatively affected Ca, Mg, and B concentrations in staminate flowers of “Wonderful”, and S concentration in staminate flowers of “Emek”.

**Figure 3:**
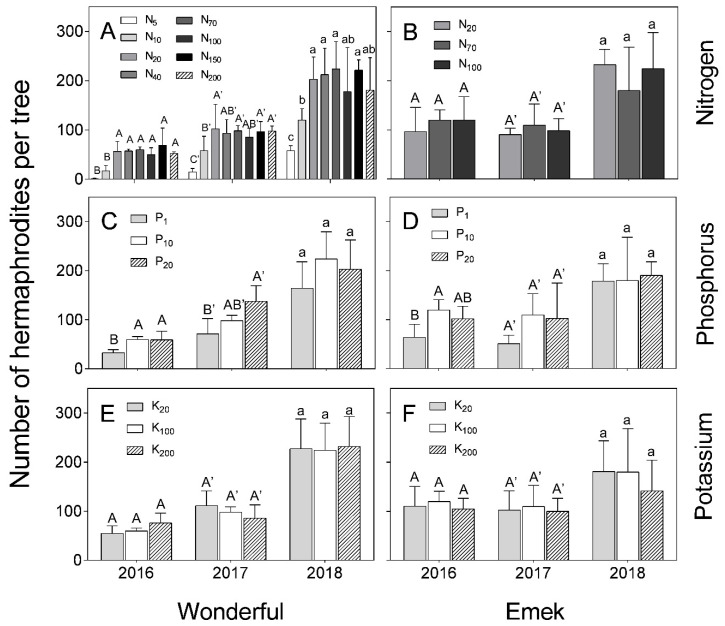
Number of hermaphroditic flowers per tree, as a function of N (**A**,**B**), P (**C**,**D**) and K (**E**,**F**) levels in the irrigation solution, in 2016–2018, for “Emek” and “Wonderful” pomegranates. Data are means ± SD of four replications. Different letters represent significant differences (*p* ≤ 0.05) between nutrient levels in the same year.

During all 3 years of the experiment, low levels of N (5 and 10 mg L^−1^) reduced hermaphrodite numbers in “Wonderful” trees (Figure 3A). In 2016, the low level of P negatively affected hermaphrodite numbers in both cultivars, and this was also the case in 2017 for “Wonderful” (Figure 3C,D). No significant differences were found between K levels in terms of hermaphrodite number, in either “Wonderful” or “Emek”, during all 3 years of the experiment (Figure 3E,F).

**Figure 4:**
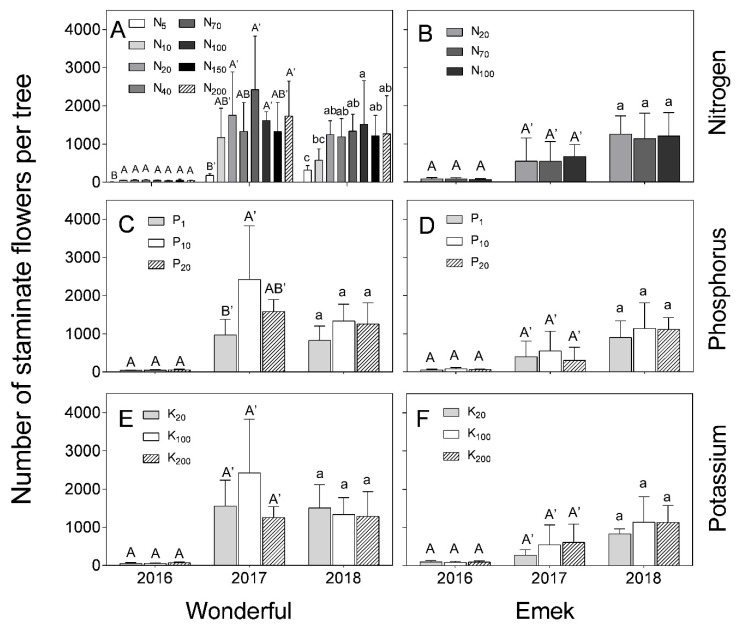
Number of staminate flowers per tree, as a function of N (**A**,**B**), P (**C**,**D**) and K (**E**,**F**) levels in the irrigation solution, in 2016–2018, for “Emek” and “Wonderful” pomegranates. Data are means ± SD of four replications. Different letters represent significant differences (*p* ≤ 0.05) between nutrient levels in the same year.

“Emek” trees did not exhibit a significant effect of N, P, or K levels on the number of staminate flowers during the 3 years of the experiment (Figure 4), but their total number increased significantly from one year to the next, with a major increase from 2016 to 2017. In “Wonderful”, there was a positive effect of N level in all 3 years of the experiment (Figure 4A), and of P level in 2017 (Figure 4C).

**Figure 5:**
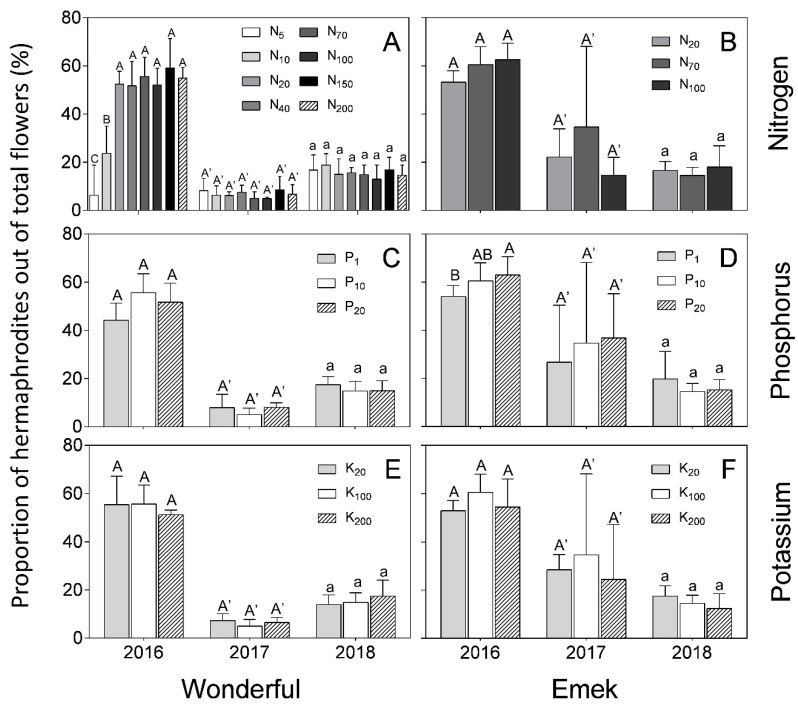
Proportion of hermaphrodites out of total flowers per tree, as a function of N (**A**,**B**), P (**C**,**D**) and K (**E**,**F**) levels in the irrigation solution, in 2016–2018, for “Emek” and “Wonderful” pomegranates. Data are means ± SD of four replications. Different letters represent significant differences (*p* ≤ 0.05) between nutrient levels in the same year.

In “Wonderful” trees, the proportion of hermaphrodites out of total flowers was 50–60% in 2016, and decreased to less than 20% in the next 2 years. In “Emek”, the proportion in 2016 was 60% in most treatments and it then decreased to less than 40% (Figure 5). In 2016, the proportion of hermaphrodites out of total flowers was positively affected by N up to 40 mg L^−1^ in “Wonderful” trees (Figure 5A), and by P in “Emek” trees (Figure 5D). No other significant effect was found for nutrient levels on this proportion, and it was relatively constant among the different fertilization levels in each year.

**Figure 6:**
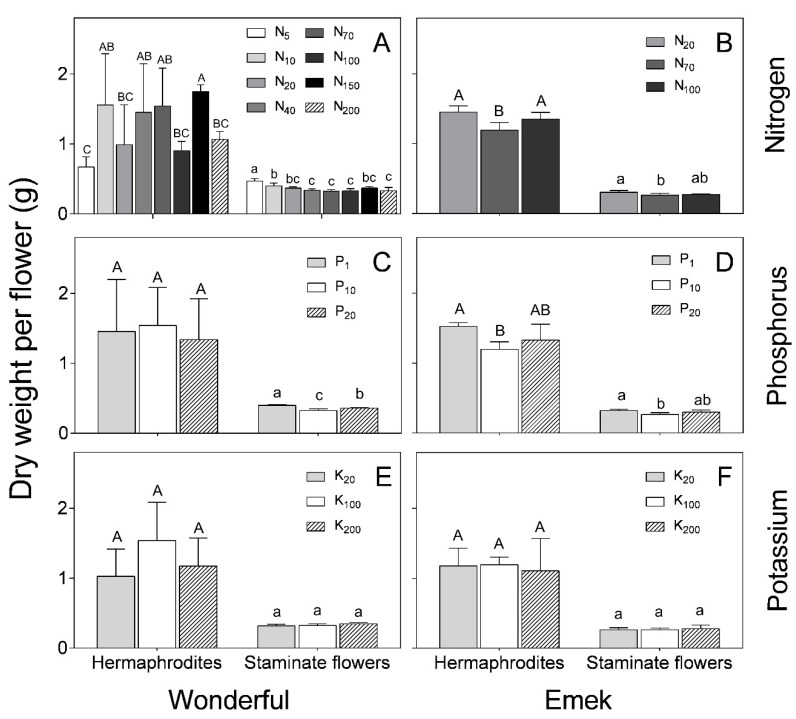
Hermaphroditic and staminate flower dry weight as a function of N (**A**,**B**), P (**C**,**D**) and K (**E**,**F**) levels in the irrigation solution, in 2018, for “Emek” and “Wonderful” pomegranates. Data are means ± SD of four replications. Different letters represent significant differences (*p* ≤ 0.05) between nutrient levels in the same year.

Hermaphrodites were characterized by a significantly higher dry weight (~1.5 g) than staminate flowers (~0.5 g) of both cultivars for all treatments (Figure 6). N and P levels affected the dry weight of both hermaphroditic and staminate flowers of “Emek” trees, with the respective medium levels resulting in the lowest dry weight (Figure 6B,D). In “Wonderful”, N and P levels negatively affected the staminate flowers (Figure 6A,C). K level had no significant effect on the dry weight of either hermaphroditic or staminate flowers, in either cultivar (Figure 6E,F).

**Figure 7:**
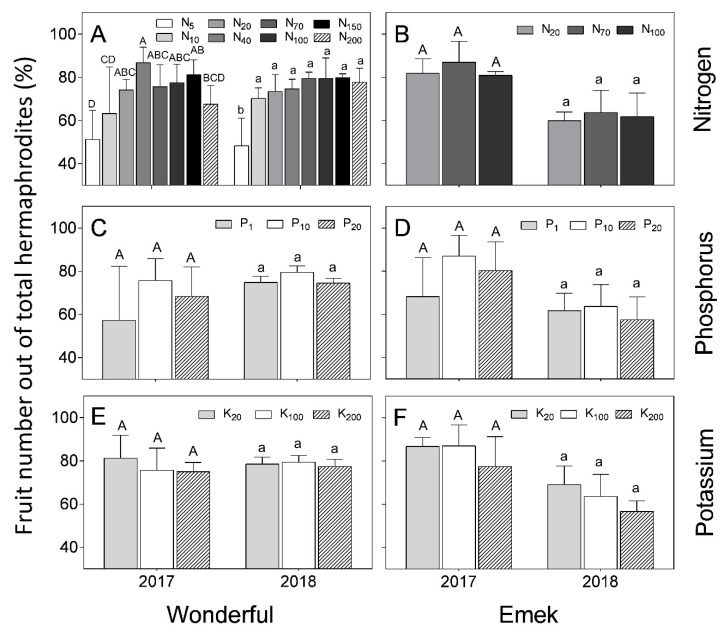
Proportion of fruit number out of total hermaphrodites per tree, as a function of N (**A**,**B**), P (**C**,**D**) and K (**E**,**F**) levels in the irrigation solution, in 2017 and 2018, for “Emek” and “Wonderful” pomegranates. Data are means ± SD of four replications. Different letters represent significant differences (*p* ≤ 0.05) between nutrient levels in the same year.

The proportion of fruit out of the total number of hermaphrodites (fruit set) was not affected in “Emek” by nutrient levels and was around 80% in 2017 and 60% in 2018 (Figure 7). In “Wonderful”, N level positively affected this index.

**Figure 8:**
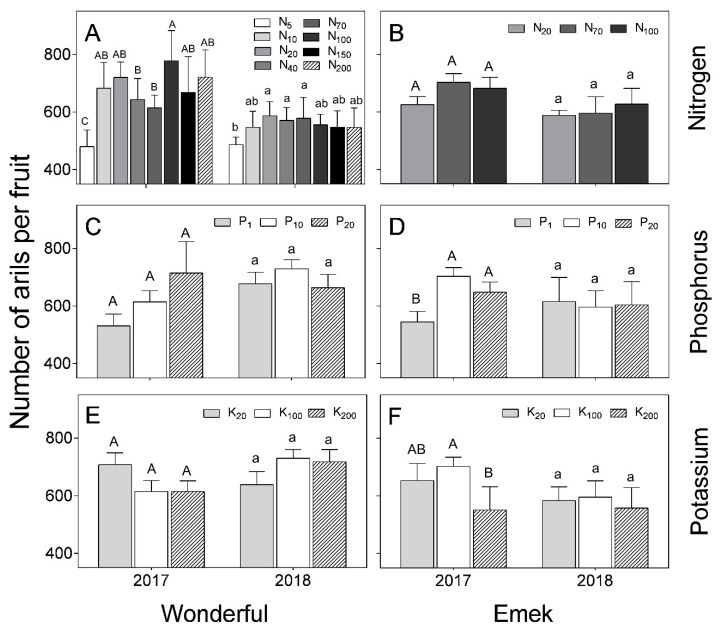
Number of arils per fruit, as a function of N (**A**,**B**), P (**C**,**D**) and K (**E**,**F**) levels in the irrigation solution, in 2017–2018, for “Emek” and “Wonderful” pomegranates. Data are means ± SD of four replications. Different letters represent significant differences (*p* ≤ 0.05) between nutrient levels in the same year.

In “Wonderful”, the number of arils per fruit decreased under low levels of N in the irrigation solution, but the response to higher levels of N was inconsistent (Figure 8). This index was generally not affected by nutrient levels. At low K levels, the number of arils per fruit was higher in 2017 than in 2018. This was also the case at the high N level. In “Emek”, the number of arils per fruit was positively affected by P levels and negatively affected by K levels in 2017.

## 3. Discussion

Nutrients are key players in plant physiology. Reproduction is a major developmental process, and nutrients are well known to participate in and affect sex expression in higher plants [20]. The first step in our experiment was to determine the macro- and microelement concentrations in pomegranate flowers as a function of N, P, and K levels in the irrigation solution. The N concentration in the flower was similar to that found in the leaves of these trees [18], refuting the assumption that the reproductive organs are a strong sink for N, as found in other species [21,22]. N level in the irrigation solution negatively affected five of the 12 nutrients tested, in both flower types of “Wonderful” trees (Figure 1). The exceptions were N itself and Mn, which responded positively to increasing levels of N. An increase in Mn concentration in response to N application has also been reported for durum wheat [23] and barley [24]. P level in the irrigation solution positively affected the P level itself, N, and Mn in the flowers. The increase in Mn in response to P application may result from the joint transport systems of these ions, as reported for the P-mobilizing strategy [25,26]. K had an inconsistent effect on flowers’ nutrient concentrations. These results reflected a clear, albeit inconsistent, physiological response of the flower to elevated nutrient levels in the irrigation solution, as found in peach [27], jasmine [28], and cotton [29].

In our experiment, nutrient levels affected the number of hermaphrodites per pomegranate tree more than the number of staminate flowers. Hermaphrodite number was positively associated with N and P levels (Figure 3). As the hermaphroditic flower is the origin of the fruit, this result is in line with the known positive effect of these macronutrients on pomegranate yield [30]. However, a previous study by our group discovered a negative effect of excess N treatments on pomegranate yield [18], meaning that many of the hermaphroditic flowers failed to set fruit, as seen in Figure 7 (in 2017) of the present study. The fact that staminate flower number was hardly affected by the mineral level (Figure 4) refutes our initial hypothesis that andromonoecy compensates for the low nutritional status of the tree. On the other hand, this hypothesis was strengthened by the fact that the proportion of hermaphrodites out of the total flowers per tree indeed decreased under low N and P irrigation levels (Figure 5). This proportion was the highest in the first year and decreased thereafter for all N, P, and K levels (Figure 5), probably due to the manual removal of hermaphrodites from the tree in 2016. We assume that the hermaphrodite number is regulated by the tree’s limited resources, and their removal enabled the redirection of resources to the young developing fruit.

As expected from their larger size, hermaphroditic flowers weighed significantly more than staminate ones, at all N, P, and K levels (Figure 6). However, it is worth noting that, in both cultivars, the heaviest staminate flowers were those under low levels of N and P, as there were fewer hermaphrodites acting as strong sinks. K level did not affect the flowers’ dry weight in either cultivar. K is considered to have a strong effect on the reproductive development of plants [31,32], but it seems that, similar to olive trees [33], its effect on pomegranate flowering is weak. A possible explanation, in line with previous results in olive [34], is that the levels tested in this study did not lead to severe K shortages, and therefore did not affect flower formation. The weak response of pomegranate to elevated K levels was also reflected in the nutrient analysis of the flowers: unlike the respective effects of N and P on their concentrations in the flowers, the K level in the irrigation solution did not affect the K concentration in the flower, except for the “Emek” staminate flowers, in which a positive effect was recorded (Figure 2). Other nutrients were indeed affected by the K level in the irrigation solution, among them N, Ca, Mn, and Mo. It is interesting to note that when only one flower type was affected by K, either positively or negatively, it was the staminate one. This result is opposite to that for N and P, where a sole flower type effect was related to hermaphrodites (Figure 2).

A significant effect of nutrient levels on fruit set was found only for the higher N treatments, mostly in 2017, in which 40 mg L^−1^ had the highest recorded fruit set (Figure 7). In 2018, no significant impact of N level on fruit set was found, except the lowest level (5 mg L^−1^), which gave the lowest result. This result is surprising because N, P, and K have been found to affect the yields of many fruit trees [35,36,37], among them pomegranate [30]. However, when the significant effect of the nutrients on hermaphrodite numbers (Figure 3) is taken into account, an impact of the specific treatments on the trees’ reproductive development was observed. We suggest that the effect of N, P, or K on yield is a consequence of their effect on tree growth, rather than directly on reproduction.

Aril (seed) number varies among pomegranate cultivars [38] and this trait is used to characterize specific varieties, due to its stability [39]. Our results supported this stability, as the aril number in “Wonderful” fruit was only affected by extreme N deficiency, and in “Emek”, by the lowest and highest levels of P and K (Figure 8). However, in 2018, aril number per fruit was not affected by nutrient levels in either cultivar, except for the lowest N level in “Wonderful”.

In summary, the various reproductive indices—the number of flowers per pomegranate tree, the proportion of hermaphrodites, fruit set, flower dry weight and the number of arils per fruit—were relatively stable and were usually affected only by an acute shortage of one of the nutritional elements. These data emphasize the pomegranate’s ability to maintain a constant fertility potential over a wide range of macroelement availability. Fertigation management in soilless-medium containers enables the frequent and continuous application of macronutrients in the water solution [40]. This system maximizes the plant’s growth and reproductive potential by bypassing the normal alternating cycles in fruit trees caused by limited nutrition. This intensive experimental system enabled us to explore the pomegranate tree’s biological response to elevated concentrations of N, P, and K in the irrigation solution. To translate these findings to horticultural recommendations, further validation experiments need to be performed in commercial orchards. The results can be used as a preliminary basis for pomegranate intensive cultivation, but further validation steps are needed.

Taken together, we revealed that N, P, and K are not the main factors affecting the reproductive development of pomegranate—either flowering or fruit set. We found that the andromonoecy trait is affected mostly by N and P deficiency, and that K has barely any influence on pomegranate reproduction.

## 4. Materials and Methods

### 4.1. Plant Material and Experimental Design

In July 2015, two-year-old “Wonderful” and “Emek” pomegranate plants were transplanted into 500-L containers filled with perlite (212, Agrekal, Israel), at the Gilat Research Center, Israel. Between July 2015 and February 2016, all trees were fertigated with 70 mg N L^−1^. During all 3 years of the experiment, the trees were fertigated daily and excessively, at rates designed to achieve 30% leaching fraction. The trees were pruned in February 2017 and 2018, after leaf drop. Trees were topped 2 m above the perlite surface, keeping only three to four main outward-growing branches. All stem-sprouting branches were removed.

Differential fertilization treatments were begun in March 2016, in two separate experiments. In one experiment, we compared three levels of N, P, and K—low, medium, and high—in both cultivars. The control fertilizer treatment, which was defined as “medium level” was 70 mg N L^−1^, 10 mg P L^−1^, and 100 mg K L^−1^. The other essential mineral nutrients were supplied with the irrigation solution as follows: Ca 20 mg L^−1^, Mg 24 mg L^−1^, S 84 mg L^−1^, Fe 500 μg L^−1^, Mn 250 μg L^−1^, Zn 125 μg L^−1^, Cu 19.0 μg L^−1^, Mo 13.5 μg L^−1^, and B 190 μg L^−1^. The low level of N was 10 mg L^−1^ for “Wonderful” and 20 mg L^−1^ for “Emek”. The high level of N was 200 mg L^−1^ for “Wonderful” and 100 mg L^−1^ for “Emek”. Other elements were as in the control treatment. P treatments were equal in both cultivars: 1 mg P L^−1^ for the low level and 20 mg P L^−1^ for the high level. K treatments were also equal in both cultivars: 20 mg K L^−1^ for the low level and 200 mg K L^−1^ for the high level.

In the other experiment, we studied the effect of eight N concentrations on “Wonderful” trees: 5, 10, 20, 40, 70, 100, 150, and 200 mg L^−1^ (90% NO3− and 10% NH4+ w/v), which included the three levels of the first experiment, to get a better response curve. The other mineral nutrients were supplied with the irrigation solution as previously described [18]. Both experiments were conducted using a randomized block design with four replications, i.e., four trees per treatment.

### 4.2. Flower Counting and Analysis

**Figure 9:**
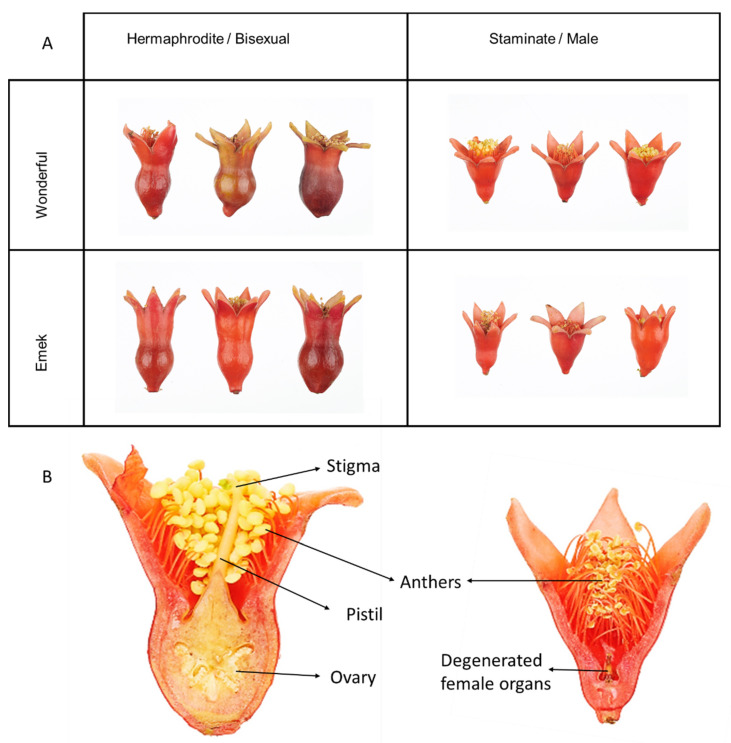
Comparison of hermaphrodite (bisexual) and staminate (functionally male) pomegranate flowers. (**A**) General view. (**B**) Cross sections.

Prior to the blooming season in each year, a plastic net was placed under each tree. The net was larger than the canopy radius. All dropped flowers were collected from the net during the entire season. The hermaphrodite and staminate flowers (Figure 9) were distinguished by style length and ovary shape, and flower numbers for each tree were recorded (fruit were considered hermaphrodites and added to their count). During the 2016 blooming season, after three fruit had set, hermaphroditic flowers were removed from the tree constantly, to reduce the fruit load and allow the tree to develop vegetatively. In the 2018 blooming season, 10 flowers of each type were sampled from each tree for mineral element analysis. N and P were analyzed using Gallery Plus (Thermo Scientific) after digesting the pulverized flowers with sulfuric acid and hydrogen peroxide. Other nutrients in the flowers were determined by digesting pulverized material with nitric acid and hydrogen peroxide, and analyzing in an ICP-OES 5100 instrument (Agilent Technologies).

### 4.3. Reproduction Indices

The 10 flowers of each type sampled from each tree during the 2018 blooming season were washed, oven-dried at 70 °C for 3 days, and their dry weight measured. Fruit set was measured for each treatment in 2017 and 2018 as the percentage of fruit per tree out of the total hermaphrodites. The arils were separated by hand from five fruits per tree. The number of arils per fruit was calculated by dividing the total aril weight by the average weight of a single aril per fruit.

### 4.4. Statistical Analyses

Data were analyzed by least squares fitting and determined as nonlinear regression functions in GraphPad Prism (GraphPad Software Inc.). Fertilizer treatments and harvest years were examined by two-way ANOVA (Table S2). The effect of fertilizer levels on andromonoecy parameters was tested by Tukey’s honest significance test (SAS Institute Inc.). *P* ≤ 0.05 was considered significant.

## Supplementary Materials

The following are available online at https://www.mdpi.com/2223-7747/9/8/963/s1, Table S1: Regression equations and statistics for Figure 1. Table S2: Two-way ANOVA analysis for the effects of fertilizer levels (Fert) and different years (Year) on the number of hermaphrodite (Herm.) and staminate flowers (Stam.), the proportion of hermaphrodites out of total flowers, fruit number from total hermaphrodites, and number of arils per fruit (Figure 3, Figure 4, Figure 5, Figure 7 and Figure 8).
