# Supplementary material for: The Effect of Macronutrient Availability on Pomegranate Reproductive Development
Source: Plants (Basel). 2020 Jul 30;9(8):963. doi: 10.3390/plants9080963 (PMC7465911; doi:10.3390/plants9080963)
Supplement: Supplementary file 1 [file plants-09-00963-s001.pdf]

Table S1. Regression equations and statistics for Figure 1.

Nutrient element	Flower type	Equation (Y = …)	R^2	*P*
Nitrogen (N)	Hermaphrodite	$19.22X/(4.941+X(1+X/1545))$	0.8515	<0.0001
	Staminate	$17.67X/(4.51+X(1+X/3908))$	0.8330	<0.0001
Phosphorus (P)	Hermaphrodite	$1.959\exp(-0.2097X)+2.755$	0.6071	<0.0001
	Staminate	$0.8179\exp(-0.1319X)+2.513$	0.2217	0.0075
Potassium (K)	Hermaphrodite	$11.95+0.1169X-0.001649X^2+5.884e\text{-}6X^3$	0.1360	0.0412
	Staminate	-	-	-
Calcium (Ca)	Hermaphrodite	$176.9\exp(-0.785X)+2.938$	0.7151	<0.0001
	Staminate	$28.31\exp(-0.5925X)+4.375$	0.2007	0.0115
Magnesium (Mg)	Hermaphrodite	$3014\exp(-1.817X)+1.183$	0.4075	0.0001
	Staminate	-	-	-
Sulfur (S)	Hermaphrodite	$1.408+0.005041X-2.348e\text{-}5X^2$	0.2908	0.0017
	Staminate	$1.859X/(1.728+X(1+X/2571))$	0.3280	0.0008
Iron (Fe)	Hermaphrodite	$33.44-0.1304X+0.001794X^2-5.292e\text{-}6X^3$	0.1531	0.0295
	Staminate	$38.5+0.1441X-0.0002098X^2-5.604e\text{-}7X^3$	0.3345	0.0007
Manganese (Mn)	Hermaphrodite	$18X/(7.959+X(1+X/3.67e153))$	0.4401	<0.0001
	Staminate	$33X/(40.26+X(1+X/2.982e104))$	0.6433	<0.0001
Copper (Cu)	Hermaphrodite	$16.17\exp(-0.1698X)+6.312$	0.8850	<0.0001
	Staminate	$8.874\exp(-0.115X)+6.787$	0.6444	<0.0001
Zinc (Zn)	Hermaphrodite	$16.39+0.08718X-0.0002934X^2$	0.3343	0.0007
	Staminate	$15.06+0.03584X-7.435e\text{-}5X^2$	0.3266	0.0008
Molybdenum (Mo)	Hermaphrodite	$2.157\exp(-0.2148X)+0.391$	0.8902	<0.0001
	Staminate	$1.715\exp(-0.2981X)+0.6665$	0.2402	0.0051
Boron (B)	Hermaphrodite	$24.76-0.06911X+0.0007359X^2-2.67e\text{-}6X^3$	0.2336	0.0059
	Staminate	-	-	-

Table S2. Two-way ANOVA analysis for the effects of fertilizer levels (Fert) and different years (Year) on the number of hermaphrodite (Herm.) and staminate flowers (Stam.), the proportion of hermaphrodites out of total flowers, fruit number from total hermaphrodites, and number of arils per fruit (Figures 3,4,5,7, and 8).

Parameters	Cultivars	Treatments	Nitrogen level			Phosphorus level			Potassium level		
			Df	F	Pr(>F)	Df	F	Pr(>F)	Df	F	Pr(>F)
Herm. number (Figure 3)	Wonderful	Fert	2	12.731	0.0002	2	5.3887	0.0117	2	0.0451	0.9560
		Year	2	55.6819	<0.0001	2	52.4464	<0.0001	2	68.2464	<0.0001
		Fert-Year	4	1.3568	0.2783	4	0.8969	0.4812	4	0.4439	0.7757
	Emek	Fert	2	0.3024	0.7418	2	2.813	0.0799	2	0.6503	0.5308
		Year	2	18.6231	<0.0001	2	17.7133	<0.0001	2	6.4844	0.0056
		Fert-Year	4	0.7777	0.5506	4	0.5805	0.6797	4	0.2051	0.9331
Stam. number (Figure 4)	Wonderful	Fert	2	2.5103	0.1023	2	4.1229	0.0289	2	1.2177	0.3136
		Year	2	16.431	<0.0001	2	25.8256	<0.0001	2	23.983	<0.0001
		Fert-Year	4	0.8294	0.5196	4	1.8081	0.1602	4	1.3286	0.2880
	Emek	Fert	2	0.1904	0.8279	2	0.5082	0.6079	2	1.4767	0.2484
		Year	2	16.0752	<0.0001	2	25.028	<0.0001	2	25.5029	<0.0001
		Fert-Year	4	0.1945	0.9389	4	0.2904	0.8813	4	0.4202	0.7924
Herm.% (Figure 5)	Wonderful	Fert	2	9.1739	0.0011	2	0.5689	0.5736	2	0.022	0.9788
		Year	2	136.7665	<0.0001	2	274.0233	<0.0001	2	222.6686	<0.0001
		Fert-Year	4	14.5183	<0.0001	4	3.0417	0.0367	4	0.6352	0.6423
	Emek	Fert	2	0.6899	0.5113	2	0.2967	0.7460	2	0.5173	0.6026
		Year	2	35.8326	<0.001	2	23.0701	<0.0001	2	23.4774	<0.0001
		Fert-Year	4	1.184	0.3430	4	0.33	0.8550	4	0.1902	0.9412
Fruit % (Figure 7)	Wonderful	Fert	2	1.683	0.2191	2	1.6145	0.2317	2	0.6079	0.5574
		Year	1	2.1089	0.1670	1	3.0415	0.1016	1	0.1834	0.6746
		Fert-Year	2	0.1444	0.8667	2	0.6524	0.5350	2	0.5091	0.6110

	Emek	Fert	2	0.77742	0.4786	2	1.8745	0.1877	2	2.5714	0.1096
		Year	1	33.4082	<0.0001	1	15.8649	0.0012	1	25.413	0.0001
		Fert-Year	2	0.2735	0.7644	2	1.5544	0.2435	2	0.1624	0.8515
Aril number (Figure 8)	Wonderful	Fert	2	0.5771	0.5735	2	2.6724	0.1017	2	0.8449	0.4490
		Year	1	16.2223	0.0011	1	0.9886	0.3358	1	15.0759	0.0015
		Fert-Year	2	2.0696	0.1608	2	2.9219	0.0848	2	3.4909	0.0569
	Emek	Fert	2	4.368	0.0320	2	3.2658	0.0665	2	3.0997	0.0747
		Year	1	25.4951	0.0001	1	6.5753	0.0216	1	8.4155	0.0010
		Fert-Year	2	0.1473	0.8643	2	2.697	0.0999	2	1.6228	0.2301
